# Validation of suitable reference genes for gene expression analysis in the halophyte *Salicornia europaea* by real-time quantitative PCR

**DOI:** 10.3389/fpls.2014.00788

**Published:** 2015-01-21

**Authors:** Xinlong Xiao, Jinbiao Ma, Junru Wang, Xiaomeng Wu, Pengbo Li, Yinan Yao

**Affiliations:** ^1^Key Laboratory of Biogeography and Bioresource in Arid Land, Xinjiang Institute of Ecology and Geography, Chinese Academy of SciencesUrumqi, China; ^2^University of Chinese Academy of SciencesBeijing, China

**Keywords:** RT-qPCR data normalization, gene quantification, housekeeping gene, halophyte, salt stress, drought stress, nitrogen stress, temperature stress

## Abstract

Real-time quantitative polymerase chain reaction (RT-qPCR), a reliable technique for quantifying gene expression, requires stable reference genes to normalize its data. *Salicornia europaea*, a stem succulent halophyte with remarkable salt resistance and high capacity for ion accumulation, has not been investigated with regards to the selection of appropriate reference genes for RT-qPCR. In this study, the expression of 11 candidate reference genes, *GAPDH* (*Glyceraldehyde 3-phosphate dehydrogenase*), *Actin*, *α-Tub* (*α-tubulin*), *β-Tub* (*β-tubulin*), *EF1-α* (*Elongation factor 1-α*), *UBC* (*Ubiquitin-conjugating enzyme*), *UBQ* (*Polyubiquitin*), *CYP* (*Cyclophilin*), *TIP41* (*TIP41-like protein*), *CAC* (*Clathrin adaptor complexes*), and *DNAJ* (*DnaJ-like protein*), was analyzed in *S. europaea* samples, which were classified into groups according to various abiotic stresses (NaCl, nitrogen, drought, cold and heat), tissues and ages. Three commonly used software programs (geNorm, NormFinder and BestKeeper) were applied to evaluate the stability of gene expression, and comprehensive ranks of stability were generated by aggregate analysis. The results show that the relatively stable genes for each group are the following: (1) *CAC* and *UBC* for whole samples; (2) *CAC* and *UBC* for NaCl stress; (3) *Actin* and *α-Tub* for nitrogen treatment; (4) *Actin* and *GAPDH* for drought stress; (5) *α-Tub* and *UBC* for cold stress; (6) *TIP41* and *DNAJ* for heat stress; (7) *UBC* and *UBQ* for different tissues; and (8) *UBC* and *Actin* for various developmental stages. These genes were validated by comparing transcriptome profiles. Using two stable reference genes was recommended in the normalization of RT-qPCR data. This study identifies optimal reference genes for RT-qPCR in *S. europaea*, which will benefit gene expression analysis under these conditions.

## Introduction

Real-time quantitative polymerase chain reaction (RT-qPCR) is one of the most powerful and reliable techniques to quantify gene expression and is widely used because of its sensitivity, accuracy and reproducibility in gene expression analysis. However, it is necessary to utilize a suitable normalization method to control for inter-sample variation, which is caused by variations in the quality of RNA samples, reverse transcription efficiency, and PCR efficiency. Using one or more stable reference genes is the most commonly applied approach to normalize RT-qPCR data (Hamalainen et al., 2001; Chen et al., 2011).

Reference genes are generally housekeeping genes that are universally expressed in all cells and whose products are necessary for cytoarchitecture or basic metabolism (Bustin, 2002; Xu et al., 2011), such as *Actin*, *Tubulin*, *EF1-α*, and *GAPDH*. However, these traditional reference genes are not always stably expressed in different cases (Glare et al., 2002; Everaert et al., 2011; Migocka and Papierniak, 2011). Therefore, it is necessary to select corresponding reference genes that are expressed at a constant level in certain cases (Jian et al., 2008; Li et al., 2013). The selection of suitable reference genes has been performed in many plant species, such as rice (Kim et al., 2003; Jain et al., 2006), wheat (Paolacci et al., 2009), barley (Burton et al., 2004), buckwheat (Demidenko et al., 2011), tomato (Expósito-Rodríguez et al., 2008), potato (Nicot et al., 2005), sugarcane (Iskandar et al., 2004), soybean (Jian et al., 2008), coffee (Barsalobres-Cavallari et al., 2009), grape (Reid et al., 2006), poplar (Brunner et al., 2004), peach (Tong et al., 2009), *Arabidopsis thaliana* (Czechowski et al., 2005; Remans et al., 2008), *Eremosparton songoricum* (Li et al., 2012) and *Lolium perenne* (Lee et al., 2010). In *Salicornia europaea*, the *α-tubulin* gene has been used as an internal control to quantify target gene expression under salt stress (Lv et al., 2011; Ma et al., 2013); however, the stability of *α-tubulin* in *S. europaea* was not verified in this previous study. To the best of our knowledge, there is no previous report on the selection of suitable reference genes for *S. europaea*.

*S. europaea*, a succulent halophyte that is distributed in coastal and inland salt marshes, can bear as much as 1000 mM NaCl in the soil (Flowers and Colmer, 2008). Interestingly, its growth significantly improves when increasing salinity to approximately 200 mM NaCl (Ozawa et al., 2007; Lv et al., 2011), while glycophytes are negatively affected by salinity above a threshold of 50 mM (Flowers and Colmer, 2008). As a salt-absorbing euhalophyte, as much as 50% of the dry weight of *S. europaea* may be salt ions (Davy et al., 2001; Ushakova et al., 2005). Therefore, this species is promising for soil desalination, which is required for the development of agriculture on salty soils and beaches. *S. europaea* also has powerful capacity for inorganic nitrogen accumulation. Webb et al. found that *S. europaea* largely absorbed inorganic nitrogen and effectively removed it from wastewater (Webb et al., 2012). We suggest *S. europaea* is an optimal model species for exploring the molecular mechanism of effective accumulation or absorption of salt ions and inorganic nitrogen. Gene expression analysis is an important approach to substantially improve our understanding of salt tolerance, salt accumulation, and inorganic nitrogen absorption. Therefore, the selection of stable reference genes for *S. europaea* is helpful for future molecular studies using RT-qPCR.

In a previous study, we RNA-sequenced *S. europaea* samples to gain insight into the molecular basis of salt adaptation by comparing digital expression profiles between a 200 mM NaCl treatment and the control treatment. Approximately 100,000 unigenes with annotations and RPKM (reads per kilobase of exon model per million mapped reads Mortazavi et al., 2008) were generated by de novo assembly and bioinformatics analysis (Ma et al., 2013). A unigene that exhibits little variation in RPKM among various transcriptomes may indicate stable expression under the tested condition. RPKM is an effective method to globally select stable reference genes by analyzing the expression profiles among various transcriptomes (Demidenko et al., 2011; Park et al., 2013). However, the transcriptome with corresponding treatment is limited; it cannot determine a suitable reference gene for other experimental conditions. Therefore, for a systematic selection of reference genes, qPCR is still the primary approach.

## Materials and methods

### Plant materials and treatment

In this experiment, the seeds of *S. europaea*, which is not an endangered or protected species and for which no specific permission is required for collection, were collected from Xinjiang in northwest China (44°14′50.2″N, 87°51′47.8″E). The seeds were sown on sand in plastic pots (3 × 3 × 5 cm) and dampened with tap water in a greenhouse with a day/night thermoperiod of 25/20°C, a photoperiod of 16 h, and a relative humidity of 50 ± 10%. After germination, seedlings were irrigated weekly with half-strength Hoagland's solution, and 4-week-old seedlings were subjected to various abiotic stresses.

#### Abiotic stress

For drought stress, the seedlings in plastic pots were not watered and were collected at 0, 1, 2, and 3 day. The water content of the sand was determined to be 23.58%, 4.58%, 0.69%, and 0.39%, respectively. For cold and heat stress, the seedlings in pots were placed at chambers at 4°C or 42°C for 0, 2, and 24 h. For salt stress, 4-week-old seedlings were carefully transferred from sand to half-strength Hoagland's solution with 200 mM or 600 mM NaCl for 0, 2, and 24 h. For nitrogen nutrition treatment (ammonium stress), the seedlings were transferred from sand to five types of nitrogen solution for 2 h, which all contained 2.5 mM CaCl_2_, 0.5 mM KH_2_PO_4_, 2 mM MgSO_4_, 0.05 mM FeSO_4_ (EDTANa_2_), 10 mM KCl, 65 μM MnSO_4_, 50 μM H_3_BO_3_, 25 μM ZnCl_2_, 2.5 μM KI, 0.5 μM Na_2_MoO_4_, 0.1 μM CuSO_4_, and 0.1 μM CoCl_2_ in addition to 0.1 mM NH_4_Cl, 10 mM NH_4_Cl, 50 mM NH_4_Cl, 0.1 mM KNO_3_, or 10 mM KNO_3_. The shoots of *S. europaea* were collected in triplicate from each treatment, immediately frozen in liquid nitrogen, and stored at −80°C.

#### Tissue

*S. europaea* is a stem succulent halophyte with vestigial leaves and tiny flowers. The young plant has fleshy cotyledons and an elongated hypocotyl. So five parts: root, hypocotyl, cotyledon, stem, and branch were collected in triplicate, quickly frozen in liquid nitrogen, and stored at −80°C.

#### Age

Whole plants were collected in triplicate at 1 day, 1 week, 2 weeks, 4 weeks, and 8 weeks after germination. These plants were immediately frozen in liquid nitrogen and stored at −80°C.

For the systematic analysis of suitable reference genes under certain conditions, cDNA samples were classified into the following eight groups: All Samples group: all 28 samples; NaCl group: samples under NaCl stress; Nitrogen group: samples under various nitrogen stress; Drought group: samples under drought stress; Cold group: samples under cold stress; Heat group: samples under heat stress; Tissue group: samples from various tissues; and Age group: samples with different age.

### Total RNA extraction and cDNA synthesis

The total RNA was extracted using an RNeasy Mini Kit (Qiagen) following the manufacturer's instructions. To avoid DNA contamination, the RNA samples were treated with an RNase-free DNase kit (Qiagen). The total RNA concentration and purity were determined using a NanoDrop 2000 Spectrophotometer. The integrity was verified by performing 1.5% agarose gel electrophoresis. The RNA samples with absorption ratios of A260/A280 = 1.9–2.1 and A260/A230 ≈ 2.0 were used for cDNA synthesis. An aliquot of 1 μg of total RNA was used for cDNA synthesis with a final volume of 20 μL using a Reversal Transcription Reagent Kit (TaKaRa) following the manufacturer's instructions.

### Candidate genes and primer design

We assessed 11 candidate reference genes that are commonly used in RT-qPCR and that have been verified as stable genes in other species. The 11 corresponding unigenes, which have credible protein annotation (Nr and Swiss-Prot databases), appropriate expression level, and a low coefficient variable (CV) of RPKM, were screened from the *S. europaea* transcriptome (Table S1). According to the sequences of the unigenes (Data Sheet 1), specific primers were designed using Primer-BLAST in NCBI (Ye et al., 2012) with the following parameters: melting temperature = 60–62°C, primer length = 19–24 nucleotides, and product size = 100–289 bp (Table 1). LinRegPCR was used to determine the primer amplification efficiency for each sample (Ramakers et al., 2003; Ruijter et al., 2009). The primer specificity was judged by melting-curve analysis and agarose gel electrophoresis of the amplification product. Furthermore, the products were sequenced (BGI, Beijing) after inserted into pMD20-T vector (TaKaRa) to confirm the primer pairs can specifically detect target genes by RT-qPCR. Each primer sequence was checked in the *S. europaea* transcriptomes using a local BLAST search to ensure detect single gene. The primer pair which matched with multiple unigenes was excluded for further analysis.

**Table 1:** **Primer sequences and amplicon characteristics for 11 candidate reference genes**.

**Unigene**	**Gene symbol**	**Gene name**	**Arabidopsis homolog locus**	**Primer: forward/reverse**	**Amplicon size (bp)**	**E (%)**	**R^2^**
Unigene17282_All	GAPDH	Glyceraldehyde 3-phosphate dehydrogenase	At1g13440	TGGCAAAGGTTAAGATCGGAATCA	289	1.845	0.998
				ACGAAGTCAGCTCCTGTGGC			
Unigene31801_All	Actin	Actin	At1g49240	TGTTGGTCGGCCTAGACACACT	228	1.862	0.999
				AATGGGGCCTCGGTAAGCAAC			
Unigene41384_All	α-Tub	α-tubulin	At5g19770	CCACCAGTGCCTTTGAGCCA	124	1.932	0.998
				GCCACAGCAGCGTTCACATC			
Unigene41909_All	β-Tub	β-tubulin	AT5g62690	ATTCAGGGTGGTCAGTGTGGAA	100	1.960	0.999
				TGGTCCCAGTGTACTTTCCTGT			
Unigene67760_All	EF1-α	Elongation factor 1-α	At1g07940	ACCCAGCTAAGGGGGCATCA	288	1.828	0.999
				CCCTCACAGCAAAACGACCGA			
Unigene40024_All	UBC	Ubiquitin-conjugating enzyme	At3g08690	GGGCCAGTCGGTGAAGACAT	136	1.915	0.998
				ATGCAACCTTTGGGGGCTTGA			
Unigene67832_All	UBQ	Polyubiquitin	At5g03240	ACGCTGGAGGTCGAAACATC	139	1.947	0.999
				TATAATCCGCCAGTGTCCTGC			
Unigene66892_All	CYP	Cyclophilin	At2g16600	GAGAAGGGTGCAGGAAGAAAGG	183	1.933	0.999
				TCCAGGTCCAGTGTGCTTC			
Unigene4197_All	TIP41	TIP41-like protein	At4g34270	AGGAAAGCTGCTGGAGAGAGTG	105	1.918	0.999
				GGAGCCTCTGGCTAATAGTGCT			
Unigene31433_All	CAC	Clathrin adaptor complexes	At1g60780	CGTGCCTTCTGATGCGACTA	166	2.028	0.998
				TGCCTCTTCACTTGTGATGCT			
Unigene19716_All	DNAJ	DnaJ-like protein	At3g44110	TGCCATCAGATCAGTGCAAGT	169	1.935	0.999
				TCTTCGTAAGCCTCTTGCTGG			

Unigenes were selected from the transcriptome of S. europaea and searched in sequences of Arabidopsis thaliana using Blastn in GenBank. The sequences of Unigenes can be found in Data Sheet 1. The mean efficiency (E) of PCR amplification and the regression coefficient (R^2^) for each primer pair were calculated by LinRegPCR Software.

### Real-time quantitative PCR

RT-qPCR was performed in 96-well optical plates with a CFX96 Real-Time PCR Detection System (Bio-Rad, USA). The reaction mixture contained 10 μL of SYBR real-time PCR premixture (BioTeke, Beijing), 4 μL of diluted cDNA (1:10), 0.5 μL of each of the forward and reverse primers (10 μM), and 5 μL of PCR-grade water in a final volume of 20 μL. The following reaction conditions were applied: 2 min at 95°C, 40 cycles of 15 s at 95°C and 30 s at 60°C, and a melting curve protocol (plates read when increased 0.5°C every 5 s from 65°C to 95°C). The melting curve verified the amplicon specificity and confirmed that there were no primer dimers. All of the samples were run with replicates, and three no-template controls (NTC) were included in every run to monitor possible DNA contamination. The threshold cycle (Ct) values, which represent the PCR cycle when a fluorescent signal reaches the threshold, were measured according to the setting of an auto-calculated baseline threshold in Bio-Rad CFX Manager software (Bio-Rad, USA).

### Stability assessment of candidate genes

Three software programs (geNorm v3.5, NormFinder v20, and BestKeeper v1) were used to statistically analyze the expression stability of 11 reference genes. For geNorm and NormFinder analyses, the mean of the Ct values were transformed into relative expression levels according to the formula E^−ΔCt^ (ΔCt = Ct value of each sample - the minimum Ct value) (Ramakers et al., 2003). The relative expression values were then imported into geNorm and NormFinder for further analysis. For BestKeeper analysis, the Ct values were used as input data. All three of the software programs were run based on the software manuals to select suitable reference genes. Three results of the stability rankings were integrated, generating a comprehensive ranking according to the mean of three rankings. To validate the reliability of the RT-qPCR data, we analyzed the expression profiles of candidate genes in RNA-seq and ranked the 11 genes according to the coefficient of variation (CV) of RPKM. The gene with the lowest CV was regarded as the most stable gene, which was double-checked by RNA-seq and RT-qPCR data in the NaCl Group experiment.

## Results

### RT-qPCR data of candidate reference genes

**Figure 1:**
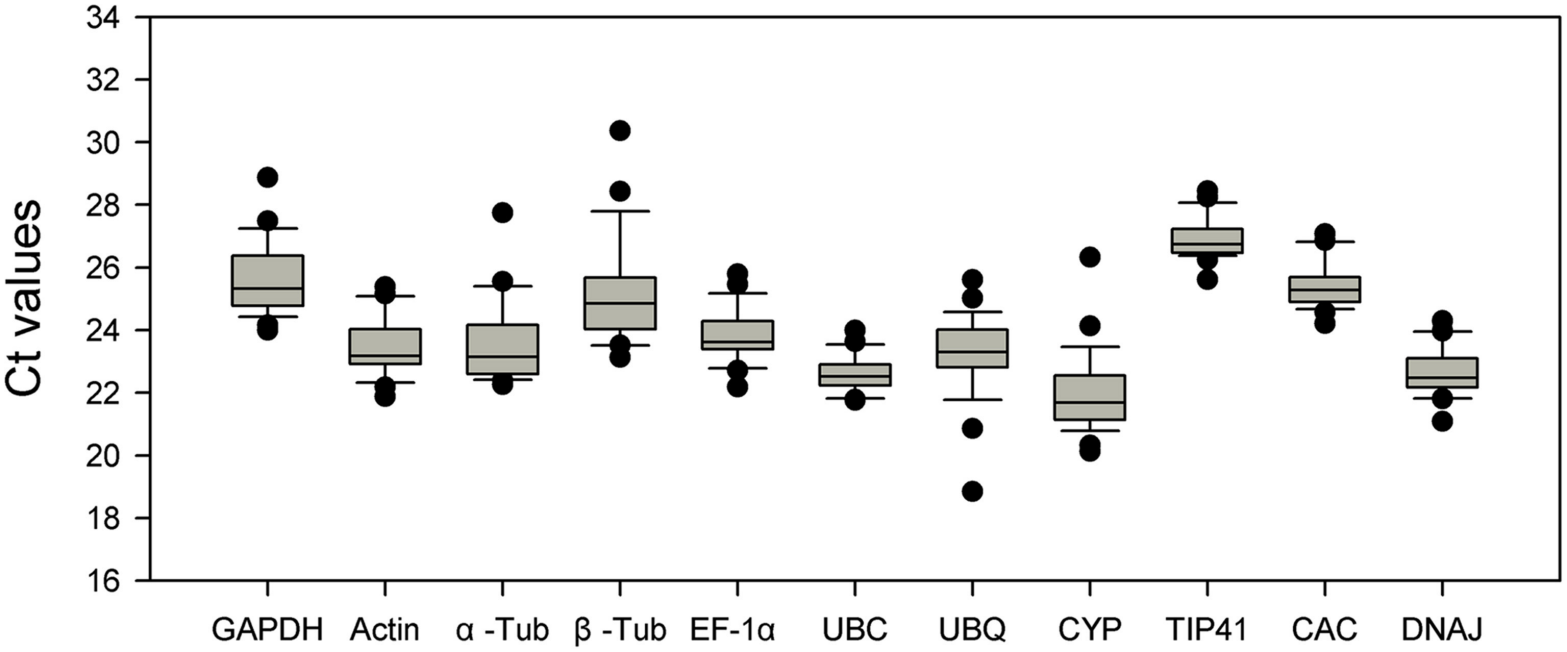
**Distribution of Ct values of candidate reference genes across all of the samples**. The Box-plot contains the mean, interquartile range, non-outlier range, and outlier.

The expression stability of candidate reference genes (*GAPDH*, *Actin*, *α-Tub*, *β-Tub*, *EF1-α*, *UBC*, *UBQ*, *CYP*, *TIP41*, *CAC*, and *DNAJ*) was assessed under various conditions, such as abiotic stress (NaCl stress, nitrogen nutrition, drought stress, heat and cold stress), in different tissues and at various stages of growth. For each gene, the mean PCR efficiency of each primer pair ranged from 1.828 to 2.028 (Table 1). Melting curve had a single-peak (Figure S1), and the gel electrophoresis of the amplification product showed a single and clear band (Figure S2). Furthermore, each sequence of the product was identical with the fragment of corresponding gene. It confirms that these primer pairs amplified unique products whose lengths and sequences are consistent with expectations. RT-qPCR was performed according to the MIQE guidelines (Bustin et al., 2009), and the Ct values in this experiment ranged from 18.84 to 30.36. The range of the Ct value represents the gene expression variation in 28 samples (Figure 1). In the box-plot, *UBC*, *CAC*, and *TIP41* showed low variability with a narrow distribution range of Ct values: 21.76–24.00, 24.20–27.06, and 25.61–28.44. *EF1-α*, *DNAJ*, *Actin*, and *GAPDH* showed medium variability, with Ct values of 22.18–25.78, 21.08–24.29, 21.88–25.38, and 24.00–28.86. However, *UBQ*, *β-Tub*, *CYP*, and *α-Tub* showed high variability, with Ct values of 18.84–25.60, 23.13–30.36, 20.12–26.32, and 22.26–27.74, and these four genes had a wider interquartile range and greater extreme outliers. *α-Tub*, *β-Tub* and *CYP* had upper outliers in the sample that was heat-treated for 24 h. This result indicates that the three genes may respond to heat stress and should not be used as reference genes under heat stress conditions. *UBQ* had lower outliers in the sample that was drought-stressed for 3 days and is therefore not a suitable reference gene for RT-qPCR under drought stress conditions. These evidently unstable genes can be easily identified in the preliminary analysis of the RT-qPCR data; however, the most stable reference gene under the various conditions should be carefully assessed using multiple methods.

### Expression stability of the candidate reference genes

To obtain a reliable dataset of the optimal reference genes for the eight groups of samples, we assessed gene stability using three software programs (geNorm, NormFinder, and BestKeeper), which are all Visual Basic application tools for Microsoft Excel. These programs are popular for the selection of suitable reference genes.

#### geNorm analysis

**Figure 2:**
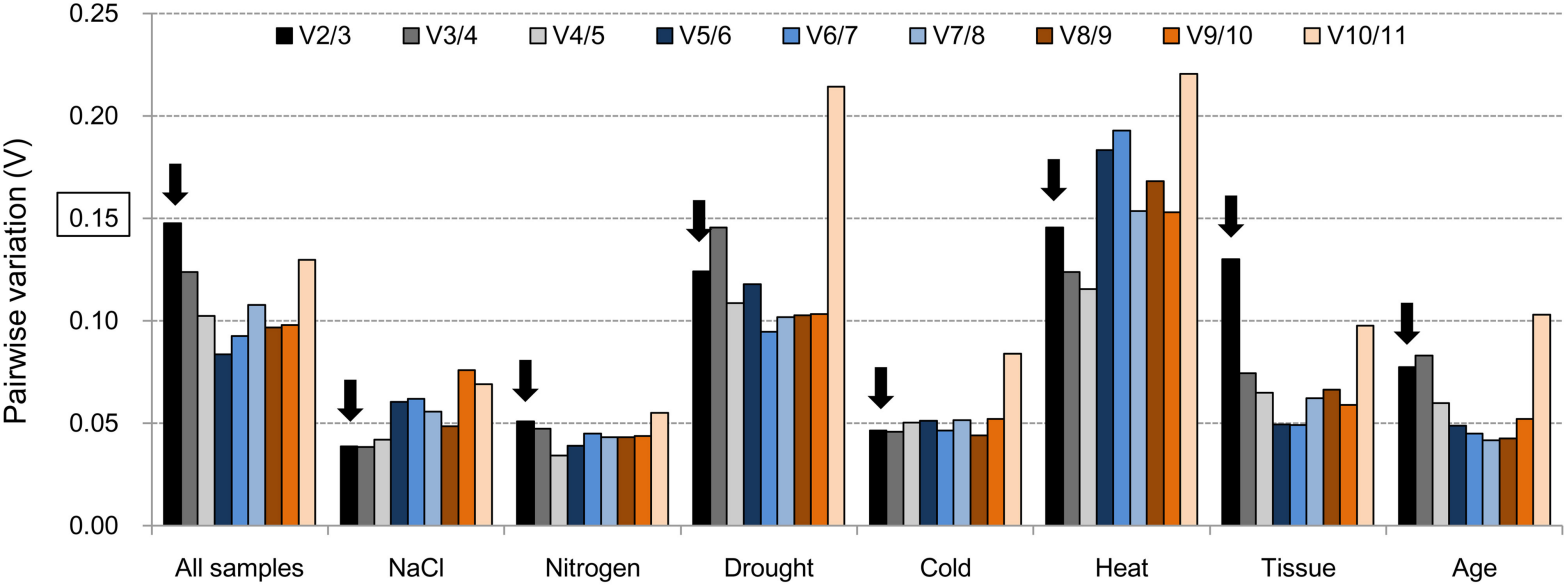
**Pairwise variation (V) values in eight groups using geNorm**. The cutoff value to determine the optimal number of reference genes for RT-qPCR normalization is 0.15. The bar marked with an arrow indicates the minimum number (n) of reference genes meeting the requirements.

The average expression stability value (*M*-value) and pairwise variation (Vn/n+1) are two parameters that are used by geNorm to assess the best reference genes. The Ct values were transformed to relative expression levels and then calculated according to the manual. The average expression stability value was calculated at each step during stepwise exclusion of the least stable reference gene until two best genes were obtained (which cannot be further compared) (Table S2). For each group, a chart of the *M*-value was generated that indicated the stability rank of the tested genes according to their average expression stability value (Figure S3). Pairwise variation (V) is an index for determining the optimal number of reference genes for accurate RT-qPCR normalization (Figure 2). A cut-off value for Pairwise variation of 0.15 was recommended by Vandesompele et al. (2002). Vn/n+1 indicates the pairwise variation between two sequential normalization factors containing an increasing number of genes. This value was calculated and compared with 0.15. If Vn/n+1 < 0.15, the optimal number of best reference genes for accurate normalization should be n; if Vn/n+1 = 0.15, this number should be n + 1. RT-qPCR data were classified as eight groups (All samples, NaCl, Nitrogen, Drought, Cold, Heat, Tissue and Age), V2/3 = 0.148, 0.039, 0.051, 0.124, 0.046, 0.145, 0.130, 0.077, respectively (Table S3). Two of best reference genes meet the requirement under these conditions for RT-qPCR normalization because the pairwise variation values of V2/3 were all less than the value 0.15: *CAC* and *EF-1α* for All samples; *UBC* and *CAC* for the NaCl group; *α-Tub* and *Actin* for the Nitrogen group; *Actin* and *GAPDH* for the Drought group; *CAC* and *UBC* for the Cold group; *DNAJ* and *TIP41* for the Heat group; *UBQ* and *UBC* for the Tissue group; and *UBC* and *EF-1α* for the Age group.

#### NormFinder analysis

Stable value (SV), which was provided by NormFinder, is a direct measure for evaluating expression variation when using reference genes for normalization. According to the rule of the software, the gene that has the lowest stable value (SV) is the most stable reference for RT-qPCR (Table S4). The results of the gene stability ranking are shown in Figure S4 for each group. The SV values of 11 genes gradually decreased, while the stability gradually increased from left to right. *UBC*, *CAC*, and *TIP41* were three best genes for the All Samples group and the NaCl group (Figures S4A,B). In the Nitrogen and Drought groups, *Actin* was the most stable gene (Figures S4C,D). *CYP* and *UBQ* were the two most stable genes in the Cold group (Figure S4E), although they were not stably expressed under most conditions, including in the All Samples group, NaCl group, Drought group, Heat group and Age group. *TIP41* and *DNAJ* were two most stably expressed in the Heat group (Figure S4F). For the Tissue group and the Age group (Figures S4G,H), *UBC* was the most stably expressed gene.

#### BestKeeper analysis

The BestKeeper program is an Excel-based tool that is similar to geNorm and NormFinder and that can evaluate gene expression stability based on repeated pair-wise correlation analysis by comparison with the BestKeeper index of each candidate gene. The coefficient of variance (CV) and the standard deviation (SD) of each gene were also calculated to evaluate the stability of each group (Table S5). The gene with the lowest CV and SD is considered to be the most stable reference (Chang et al., 2012). Because up to 10 genes can load in BestKeeper, the 10 most stable genes that were recommended by geNorm and NormFinder were imported into BestKeeper for stability analysis. The stability of the remaining one gene in each group was ranked according to the result of geNorm. As shown in Figure S5, the two most stable genes for each group were: *TIP41* and *UBC*; *TIP41* and *CAC*; *TIP41* and *UBC*; *TIP41* and *Actin*; *α-Tub* and *UBC*; *UBQ* and *CAC*; *UBQ* and *UBC*; *Actin* and *UBC*.

### Comprehensive ranking of expression stability

**Figure 3:**
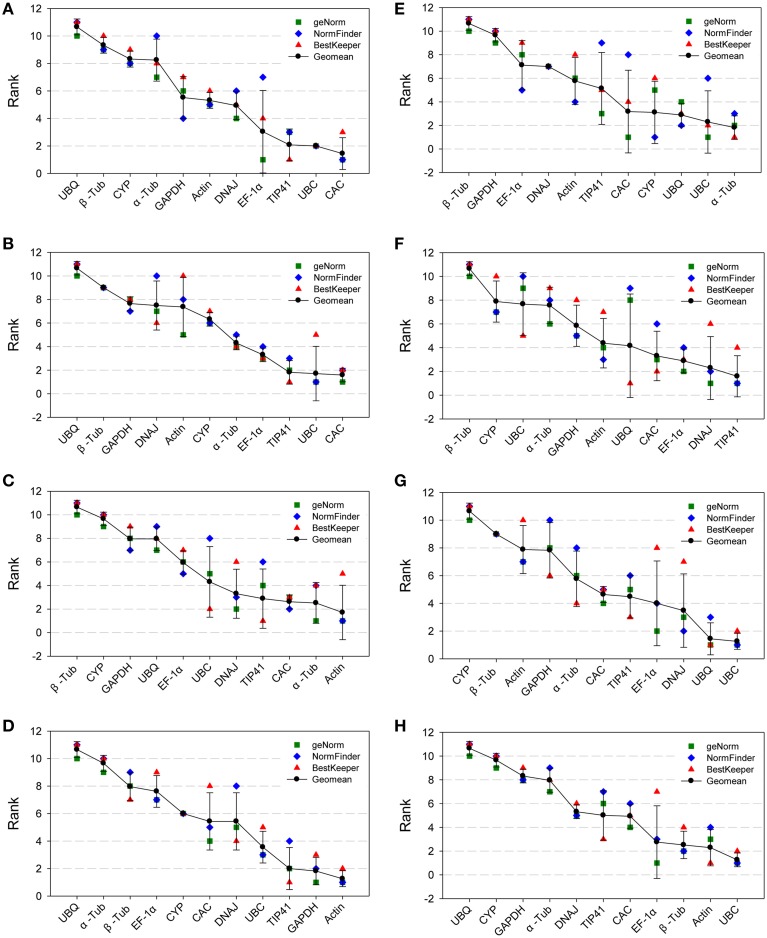
**Aggregation of three rankings**. The comprehensive ranking of candidate genes was calculated by the geometric mean of three types of rankings (geNorm, NormFinder and BestKeeper) in each group. **(A)** All Samples group; **(B)** NaCl group; **(C)** Nitrogen group; **(D)** Drought group; **(E)** Cold group; **(F)** Heat group; **(G)** Tissue group; **(H)** Age group.

To obtain clear results for the most stable reference genes as recommended by the three methods according to the RefFinder approach (Stajner et al., 2013), we calculated the geometric mean of three corresponding rankings for each candidate gene. This represented the comprehensive ranking of the gene expression stability (Figure 3 and Table S6). *UBC* and *CAC* were the two most stable genes in the All Samples group and NaCl group (Figures 3A,B) according to the comprehensive ranking. *Actin* comprehensively ranked first in the Nitrogen group and Drought groups (Figures 3C,D). Under cold treatment (Figure 3E), *α-Tub* was the best reference gene, while it was unstably expressed under heat treatment. For the Heat group (Figure 3F), *TIP41* and *DNAJ* were the two most stable genes. For both the Tissue group and the Age group (Figures 3G,H), *UBC* was the most stable gene, and the second most stable genes were *UBQ* and *Actin*. *UBQ*, *β-Tub*, and *CYP* were three most unstable genes in the All Sample group. The expression of *UBQ* was extremely unstable under NaCl stress and drought stress. *β-Tub* was unstably expressed in the majority of tested groups, especially in the Nitrogen group, Cold group and Heat group. *CYP* was the most unstable gene in the Tissue group and was unstable under most conditions.

### Validation of RT-qPCR results

**Figure 4:**
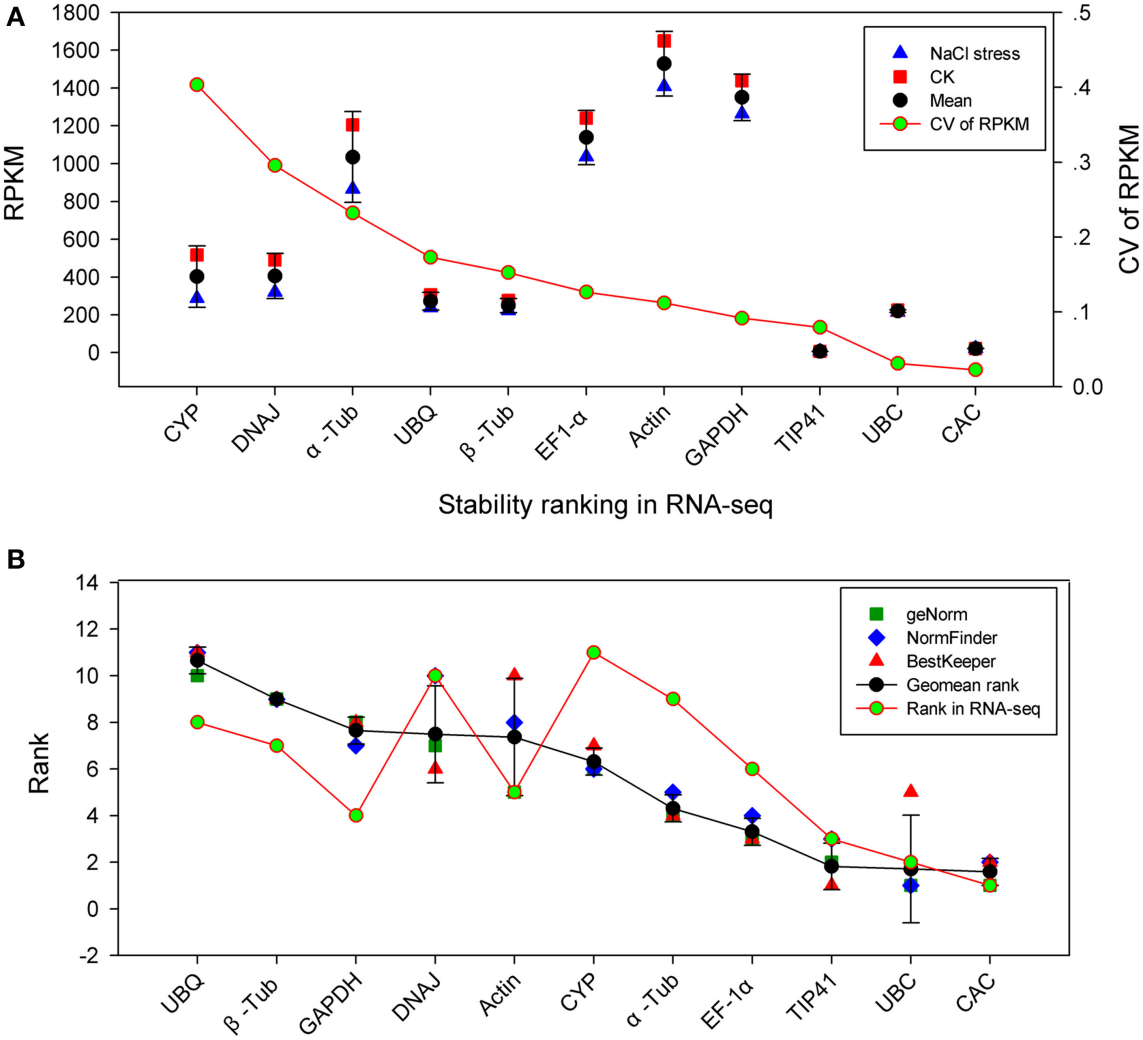
**Validation of RT-qPCR results through comparison with RNA-seq expression profiles. (A)** Stability ranking of candidate genes by CV of RPKM in RNA-seq. The gene with lower CV indicates more stable expression. **(B)** Correlation analysis between ranking of NaCl group by RT-qPCR and the ranking of RNA-seq. CK: NaCl-free; CV: coefficient of variation; RPKM: reads per kilobase of exon model per million mapped reads.

The most stable reference genes under various conditions were carefully screened through RT-qPCR experiments and scientific analysis (geNorm, NormFinder, BestKeeper, and a final comprehensive analysis). To validate this result, the stability of the candidate genes in RT-qPCR was compared with RNA-seq-based gene expression profiling. *S. europaea* samples with 200 mM NaCl stress and control treatment were mapped, and their unigenes were quantified by RNA-seq as in our previous study (Table S1). RPKM represents the expression quantities of the unigenes, and the coefficient of variation (CV) of RPKM represents the variability in gene expression. In Figure 4A, *CYP*, *DNAJ*, *α-Tub*, *UBQ*, and *β-Tub* showed a high CV value, indicating these genes were not stable genes. In contrast, *CAC*, *UBC* and *TIP41* had a lower CV, indicating that they were more stable under NaCl stress. We compared the ranking of gene stability in the NaCl group with the ranking of these genes in the RNA-seq data (Figure 4B). To some extent, the two types of rankings were consistent and had a positive correlation coefficient of *r* = 0.59. *CAC*, *UBC*, and *TIP41*, which were three most stable genes in NaCl group, were show relative stable expression under NaCl stress through transcriptome analysis. Unstable genes, such as *UBQ*, *β-Tub*, and *DNAJ*, also had similar rank in NaCl group and transcriptome analysis. The result of NaCl group was verified by RNA-seq data, demonstrating the quality of the RT-qPCR data and increasing the reliability of the remaining results in this study.

### The effects of optimizing reference genes for relative quantification

**Figure 5:**
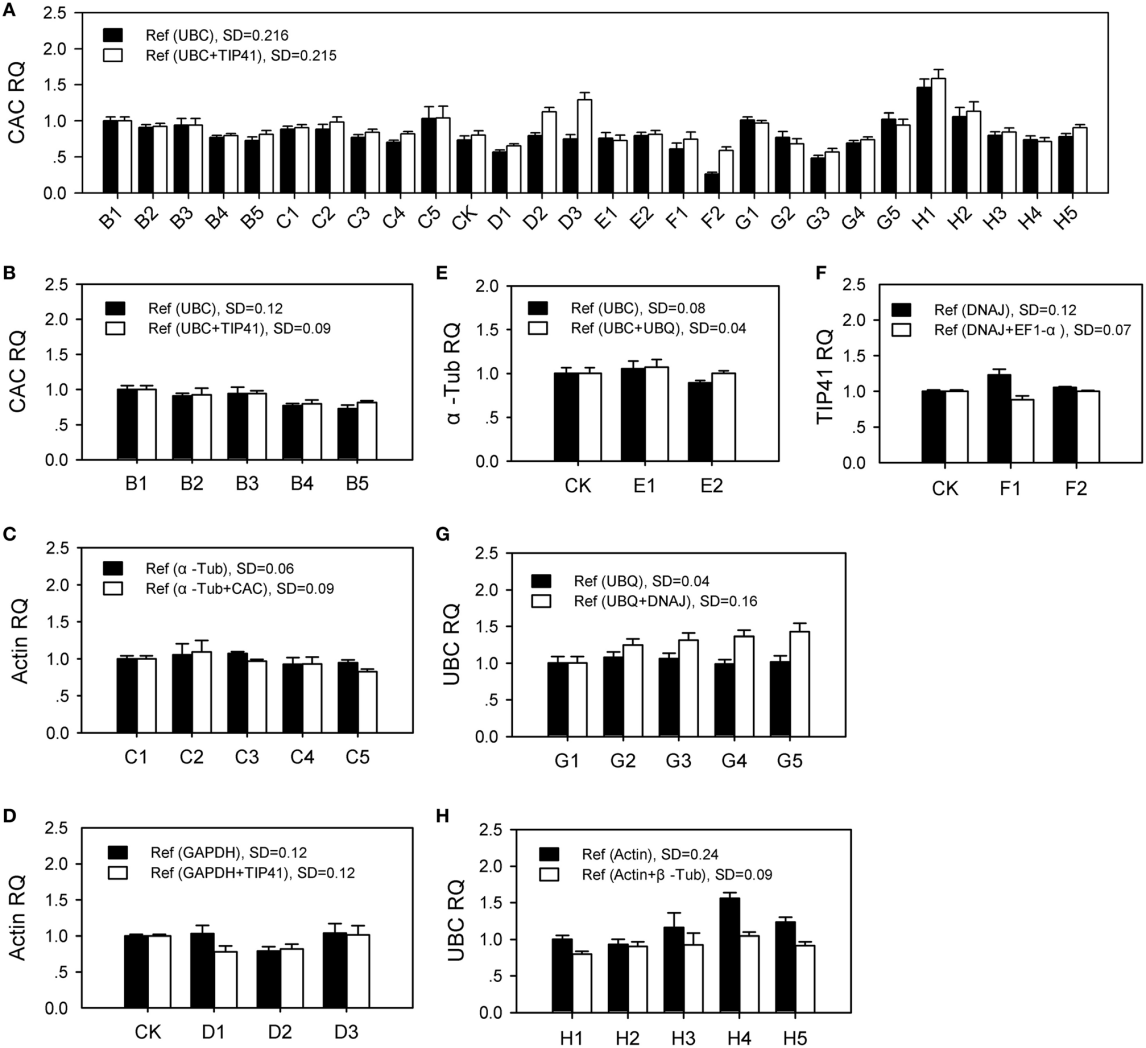
**The effect of two reference genes vs. single gene to normalize the most stable gene in RT-qPCR**. SD: standard deviation. Ref: reference gene. RQ: gene relative quantity. B1–B5: samples under NaCl stress (200 mM for 0 h, 2 h, and 24 h; 600 mM for 2 h and 24 h); C1–C5: samples under different nitrogen nutrition (0.1, 10, and 50 mM NH^+^_4_; 0.1 and 10 mM NO^−^_3_); CK: control sample; D1–D3: samples under drought stress for 1 day, 2 day, and 3 day; E1, E2: samples under cold stress for 2 h and 24 h; F1, F2: samples under heat stress for 2 h and 24 h; G1–G5: root, hypocotyl, cotyledon, stem, and branch; H1–H5: different ages at 1 day, 1 week, 2 weeks, 4 weeks, and 8 weeks. **(A)** All Samples group; **(B)** NaCl group; **(C)** Nitrogen group; **(D)** Drought group; **(E)** Cold group; **(F)** Heat group; **(G)** Tissue group; **(H)** Age group.

Multiple stable housekeeping genes were recommend in qPCR data analysis by using their geometric mean (Vandesompele et al., 2002). As shown in Figure 5, in which the most stable gene (ranked first in Figure 3) was considered target gene, single gene (ranked second) or two genes (ranked second and third, respectively) were used as reference genes for RT-qPCR relative quantification (2^−ΔΔCt^ method Livak and Schmittgen, 2001) in each group (Table S7). The target gene should be stably expressed with an expected relative quantity value 1 under corresponding conditions, which based on an assumption that target gene and reference genes are all expressed at constant level. For specific conditions, corresponding target gene was relatively stable expression, while the quantitative result in All samples group was not satisfactory. Furthermore, it generally obtains more accurate result when using two internal control genes, in which the target genes showed lower expression SD. Two stable reference genes were recommended using in RT-qPCR data analysis for the seven groups. However, the relative stable genes selected in this study could not be qualified as normalization of qPCR data in all samples.

## Discussion

*S. europaea*, which accumulates ions, is one of the most salt-tolerant halophytes worldwide. The need for suitable reference genes is urgent for RT-qPCR detection of gene expression regarding the salt resistance and ion absorption processes because we have little information on the stable reference genes in this species.

In our study, three software programs (geNorm, NormFinder, and BestKeeper) were used to evaluate the stability of candidate genes. The three rankings of the reference genes were nearly identical in NaCl group (Figure 3B), Drought group (Figure 3D), and Age group (Figure 3H). However, because of the different algorithms, the rankings provided by the three programs were not completely identical. For example, in the All Samples group, *CAC* was ranked first by geNorm and NormFinder, while it was ranked third by BestKeeper. Furthermore, in the All samples group and Age group, the most stable genes in geNorm (Figures S3E,H), such as *EF1-α*, was ranked at a medium position or even a bottom position in NormFinder (Figures S4A,G) or BestKeeper (Figures S5A,G). Regarding the divergent results, unfortunately, there has not yet been a report on the comparison of reliability among the three programs. Therefore, referring to a previous study (Stajner et al., 2013), the geometric mean of three rankings was calculated to generate a clear comprehensive ranking for each gene (Figure 3). A lower mean of rankings indicates that the gene is more stable, and more narrow error bars indicate that the result is more reliable. The comprehensively ranked first gene in NaCl group, Drought group Tissue group and Age group (*CAC*, *Actin*, *UBC*, and *UBC*, respectively) have lower geometric mean and narrower error bars because they all ranked first or second in geNorm, NormFinder, and BestKeeper. It is more reliable that the ranked first gene was the relative most stable gene in corresponding group.

In our study, the stability of the 11 candidate reference genes was analyzed in eight groups. Different groups of samples had their own optimal reference genes. As shown in Figure 3, *α-Tub* was more stably expressed in the Cold group, but it was less stable than the other genes in the Drought group and Heat group. *Actin* was the most stable gene in the Nitrogen group and Drought group, but it was not the best gene in the other groups. Therefore, it is necessary to confirm the expression stability of the reference genes under specific conditions.

Of the three top genes in the All Samples group, *UBC* was ranked first in the Tissue group and Age group. The strong stability of *UBC* in *S. europaea* was consistent with the result in *Platycladus orientalis* (Chang et al., 2012); however, *UBC* is not satisfactory for RT-qPCR normalization in different tissues of bamboo (Fan et al., 2013). *CAC*, a clathrin adaptor complex gene, was ranked first in the All Samples group and NaCl group. In other species, *CAC* was also highly stable under salt stress in *Cucurbita pepo* (Obrero et al., 2011), while it was the least stable gene in *P. orientalis* (Chang et al., 2012). *TIP41* was a stable gene in the Nitrogen group, NaCl group, Heat group and Drought group. This gene was also expressed stably under abiotic stress in *Brassica juncea* (Chandna et al., 2012), at different developmental stages of olive plants and in various tissues of bamboo (Fan et al., 2013; Resetic et al., 2013). However, this gene was not a suitable reference in *Coffea arabica* under nitrogen starvation, salt stress or heat stress (De Carvalho et al., 2013). The results are diverse in various studies that evaluated reference genes in different plant species. The tested genes, which were labeled with the same names, belonging to multigene families could be one of the sources of variations in the published studies (Oakley et al., 2007). In fact, the primer sequence should be the identification of the selected reference gene rather than its name.

By RNA-seq, we can obtain a large amount of transcript information as well as expression profiling of thousands of genes (Wang et al., 2009). In the absence of a sequenced genome, the primer pairs of RT-qPCR in this study were designed based on assembled unigenes which represented full-length or part of transcripts. The amplification specificity of the primers is essential for detecting the expression of target gene by RT-qPCR (Guenin et al., 2009). Although the traditional methods that detect melting-curve, agarose gel electrophoresis, and product sequencing can confirm the unique PCR product, these methods cannot judge the primer pair detects single gene, because of the gene duplication or transposition in plant genomes. Therefore, the specificity of the primers should be checked against the transcriptomes. Only the primer that matched with single unigene was used for gene stability evaluation through RT-qPCR. RT-qPCR is often used to validate the transcriptome profiling expression (Ma et al., 2013; Yao et al., 2014). In this study, to validate the results of RT-qPCR, we compared the result with RNA-seq data whose samples under same condition. The two results supported each other, as they had a significant positive correlation coefficient. Therefore, the results of this experiment are credible. Furthermore, it is helpful using multiple stable reference genes for accurate normalization of RT-qPCR data. Appropriate amount of stable reference genes are essential in specific RT-qPCR experiment.

## Conclusion

The selection of suitable reference genes is a prerequisite to quantifying gene expression by RT-qPCR. We evaluated 11 candidate reference genes for the normalization of RT-qPCR in *S. europaea* samples of different tissues, ages, and plants subjected to various treatments. The stability of the genes was analyzed through three commonly used applications, and their results were integrated into a comprehensive stability ranking based on the geometric mean. For the study of abiotic stresses (including salt stress, nitrogen nutrition (ammonium stress), drought stress, cold stress, and heat stress), we recommend *CAC* and *UBC*, *Actin* and *α-Tub, Actin*, and *GAPDH*, *α-Tub*, and *UBC*, and *TIP41* and *DNAJ*, respectively, to normalize RT-qPCR data, while the least stable genes, which should not be used in the corresponding conditions, were *UBQ* and *β-Tub*, *β-Tub* and *CYP*, *UBQ* and *α-Tub*, *β-Tub* and *GAPDH*, and *β-Tub* and *CYP*, respectively. For the study of gene expression in different tissues of *S. europaea*, *UBC* and *UBQ* are recommended as the stable reference genes. For the study of gene expression in various developmental stages of *S. europaea*, *UBC* and *Actin* are the best reference genes. Two stable reference genes are recommended using in these conditions. The reliability of these results was enhanced through comparison between part RT-qPCR result and RNA-seq data, and the selected reference genes can significantly reduce errors in genes quantification. This study will benefit future studies of gene expression in *S. europaea* using RT-qPCR.

### Conflict of interest statement

The authors declare that the research was conducted in the absence of any commercial or financial relationships that could be construed as a potential conflict of interest.

## Supplementary material

Figure S1 **Melting curves with a single melt peak in qPCR**. To confirm primer specificity and no primer dimers, qPCR melting curves of 11 primers were detected when the primer standard curves were generated using a series of 10-fold cDNA dilution.

Figure S2 **Agarose gel electrophoresis of qPCR products**. qPCR reactions were performed with each primer pair and a pooled cDNA template. Amplified fragments were separated by 1.2% agarose gel electrophoresis. Both DNA markers have bands of 2000 bp, 1000 bp, 750 bp, 500 bp, 250 bp, and 100 bp (ranking from high to low).

Figure S3 **Average expression stability values (M) of 11 genes in eight groups by geNorm**. A lower *M*-value indicates more stable expression.

Figure S4 **Stability ranking of candidate genes by NormFinder**. A lower Stable Value indicates more stable expression. **(A)** All Samples group; **(B)** NaCl group; **(C)** Nitrogen group; **(D)** Drought group; **(E)** Cold group; **(F)** Heat group; **(G)** Tissue group; **(H)** Age group.

Figure S5 **Stability ranking of candidate genes by BestKeeper**. The gene with lower CV and SD shows more stable expression. CV: coefficient of variation; SD: standard deviation. **(A)** All Samples group; **(B)** NaCl group; **(C)** Nitrogen group; **(D)** Drought group; **(E)** Cold group; **(F)** Heat group; **(G)** Tissue group; **(H)** Age group.

Table S1 **Selected Unigenes from the transcriptome of *Salicornia europaea***. The unigenes with appropriate expression quantities and highly reliable annotation were selected to evaluate the stability of gene expression.

Table S2 **geNorm ranking of candidate genes**. *M*-values: Average expression stability values. Gene stability was ranked according to the *M*-values.

Table S3 **Data of Vn/n+1 to determine the optimal number of reference genes in geNorm compared to a threshold of 0.15**.

Table S4 **NormFinder ranking of candidate genes**. SV: stable value. Gene stability was ranked according to the SV.

Table S5 **BestKeeper ranking of candidate genes**. A total of 10 stable reference genes recommended by geNorm and NormFinder were analyzed in BestKeeper. The stability of genes was ranked according to CV and SD, and the remaining two least stable genes were ranked according to the result of geNorm. CV: variable coefficient; SD: standard deviation. NA: no analysis.

Table S6 **Comprehensive stability ranking of candidate genes based on the results of the three software programs**. The comprehensive ranking of each gene was calculated by the geometric mean of the rankings in geNorm, NormFinder, and BestKeeper. Ranked by the mean values.

Table S7 **Relative quantities of stable genes in corresponding groups**. The most stable gene and the least stable gene were used as the reference genes for RT-qPCR in corresponding groups.

Data Sheet 1 **Sequence of unigenes in the transcriptome of *Salicornia europaea***. Specific primers for RT-qPCR were designed based on these sequences in Test S1. Amplification products were verified by sequencing.
