# Supplementary figures and images for: Validation of suitable reference genes for gene expression analysis in the halophyte Salicornia europaea by real-time quantitative PCR
Source: Front Plant Sci. 2015 Jan 21;5:788. doi: 10.3389/fpls.2014.00788 (PMC4300904; doi:10.3389/fpls.2014.00788)

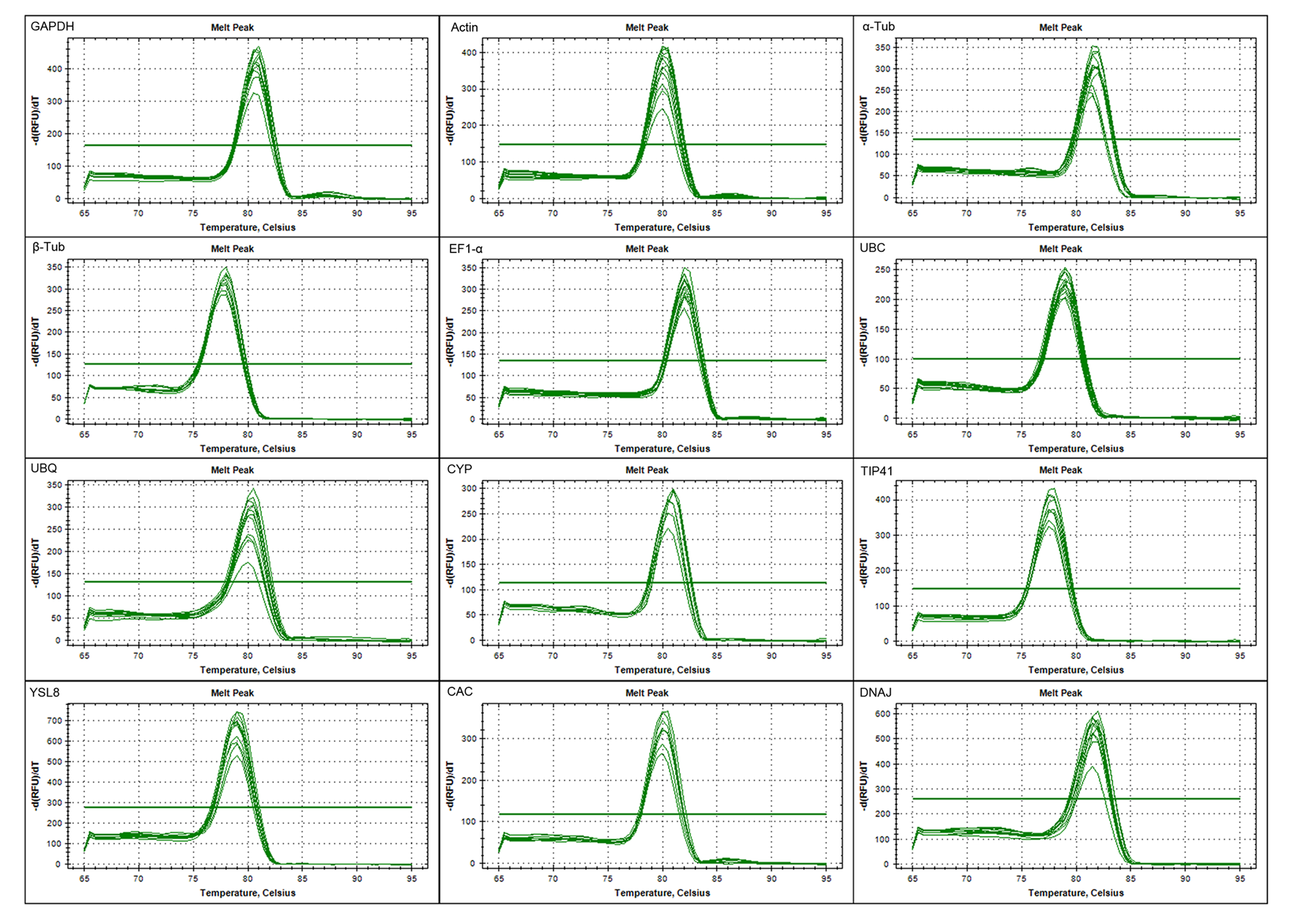

Supplement: Figure S1 — Melting curves with a single melt peak in qPCR. To confirm primer specificity and no primer dimers, qPCR melting curves of 11 primers were detected when the primer standard curves were generated using a series of 10-fold cDNA dilution. [file Image1.TIF]

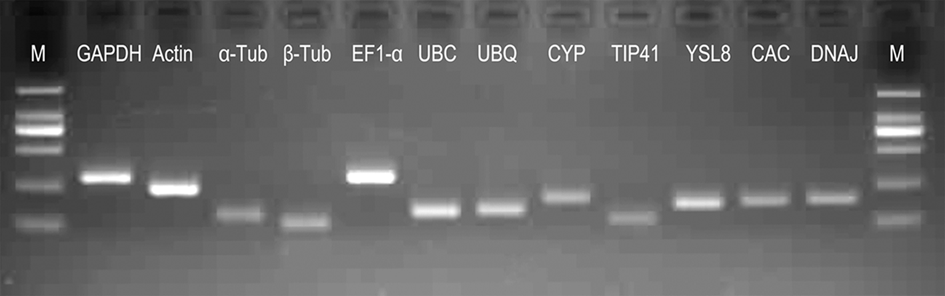

Supplement: Figure S2 — Agarose gel electrophoresis of qPCR products. qPCR reactions were performed with each primer pair and a pooled cDNA template. Amplified fragments were separated by 1.2% agarose gel electrophoresis. Both DNA markers have bands of 2000 bp, 1000 bp, 750 bp, 500 bp, 250 bp, and 100 bp (ranking from high to low). [file Image2.TIF]

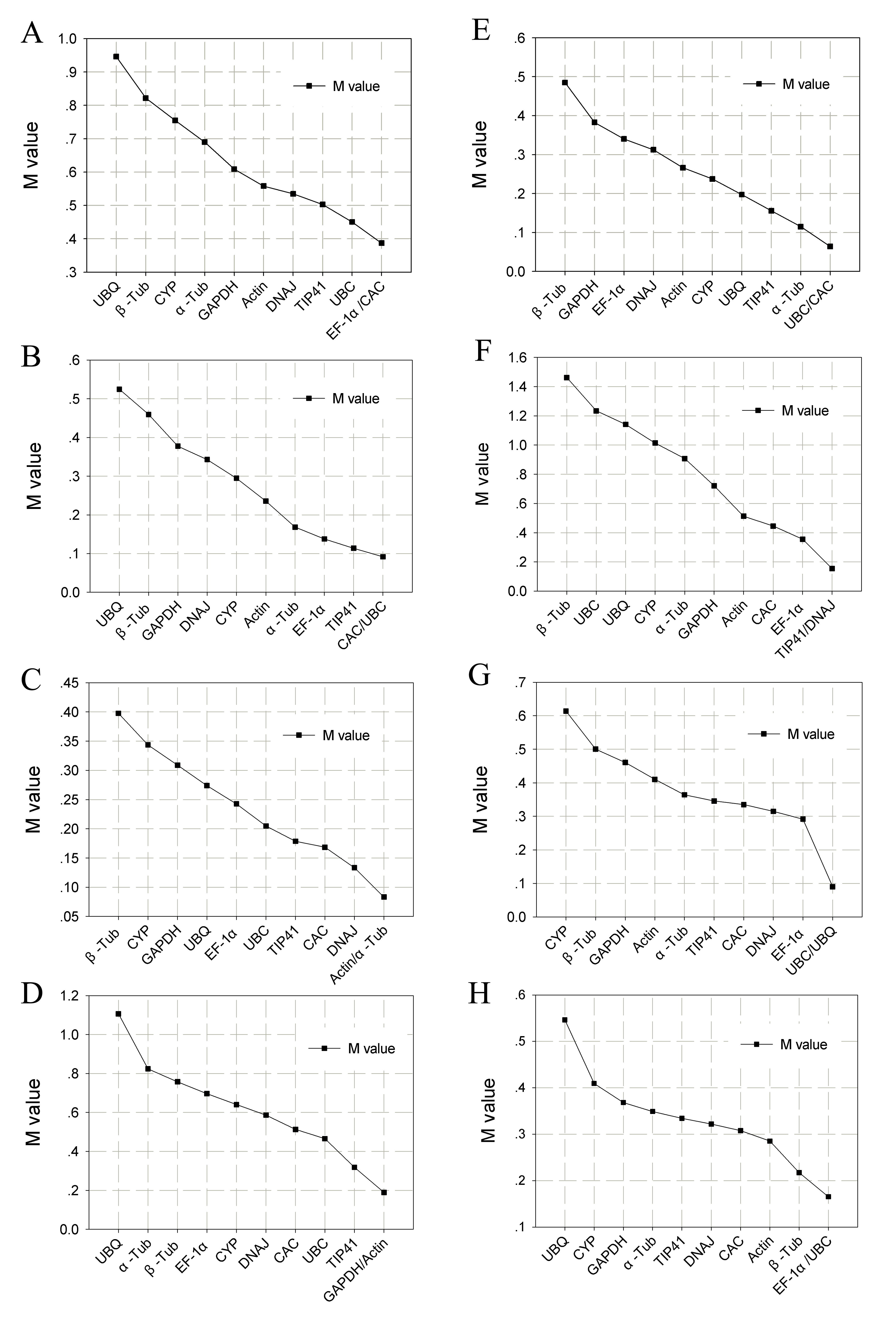

Supplement: Figure S3 — Average expression stability values (M) of 11 genes in eight groups by geNorm. A lower M-value indicates more stable expression. [file Image3.TIF]

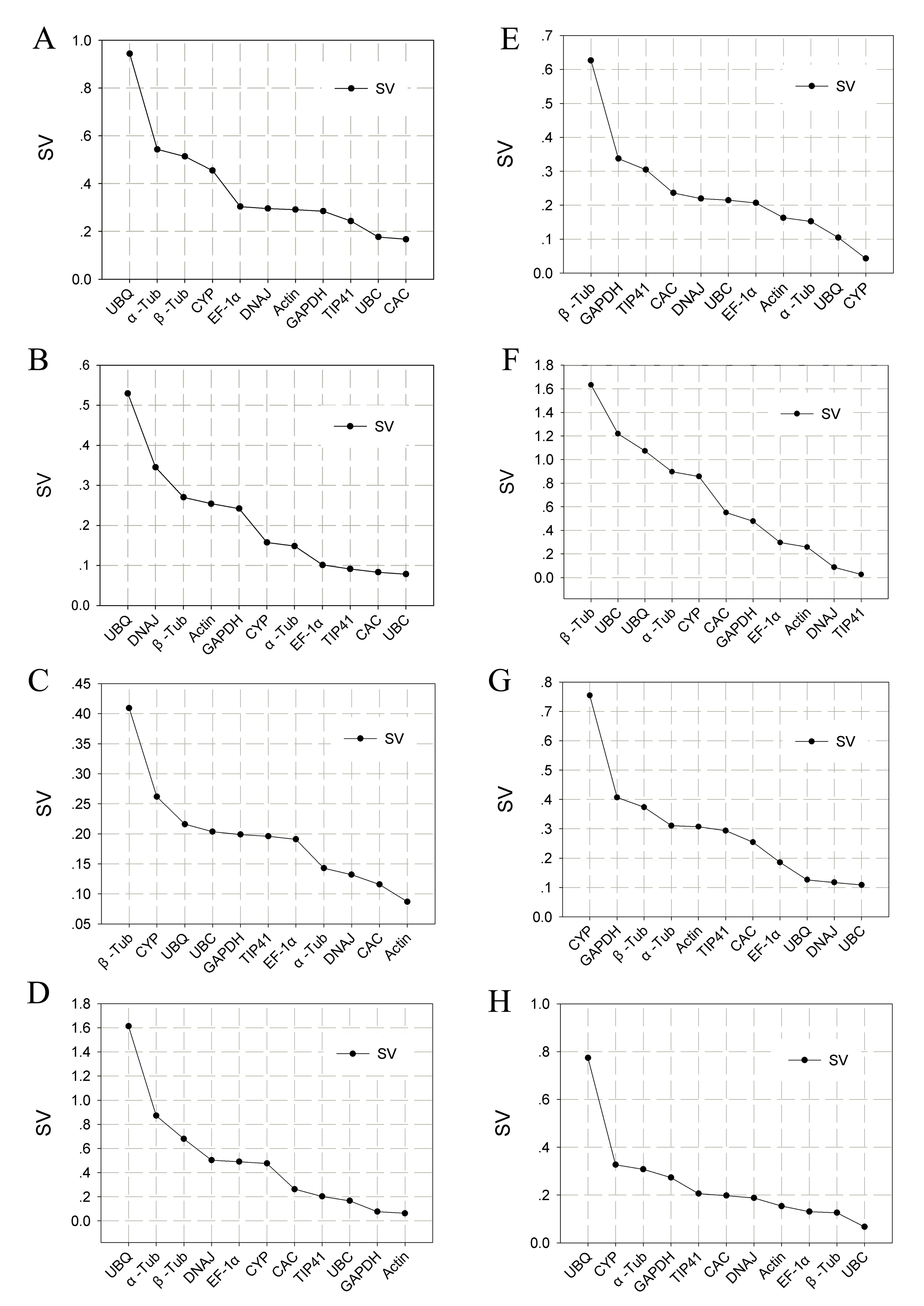

Supplement: Figure S4 — Stability ranking of candidate genes by NormFinder. A lower Stable Value indicates more stable expression. (A) All Samples group; (B) NaCl group; (C) Nitrogen group; (D) Drought group; (E) Cold group; (F) Heat group; (G) Tissue group; (H) Age group. [file Image4.TIF]

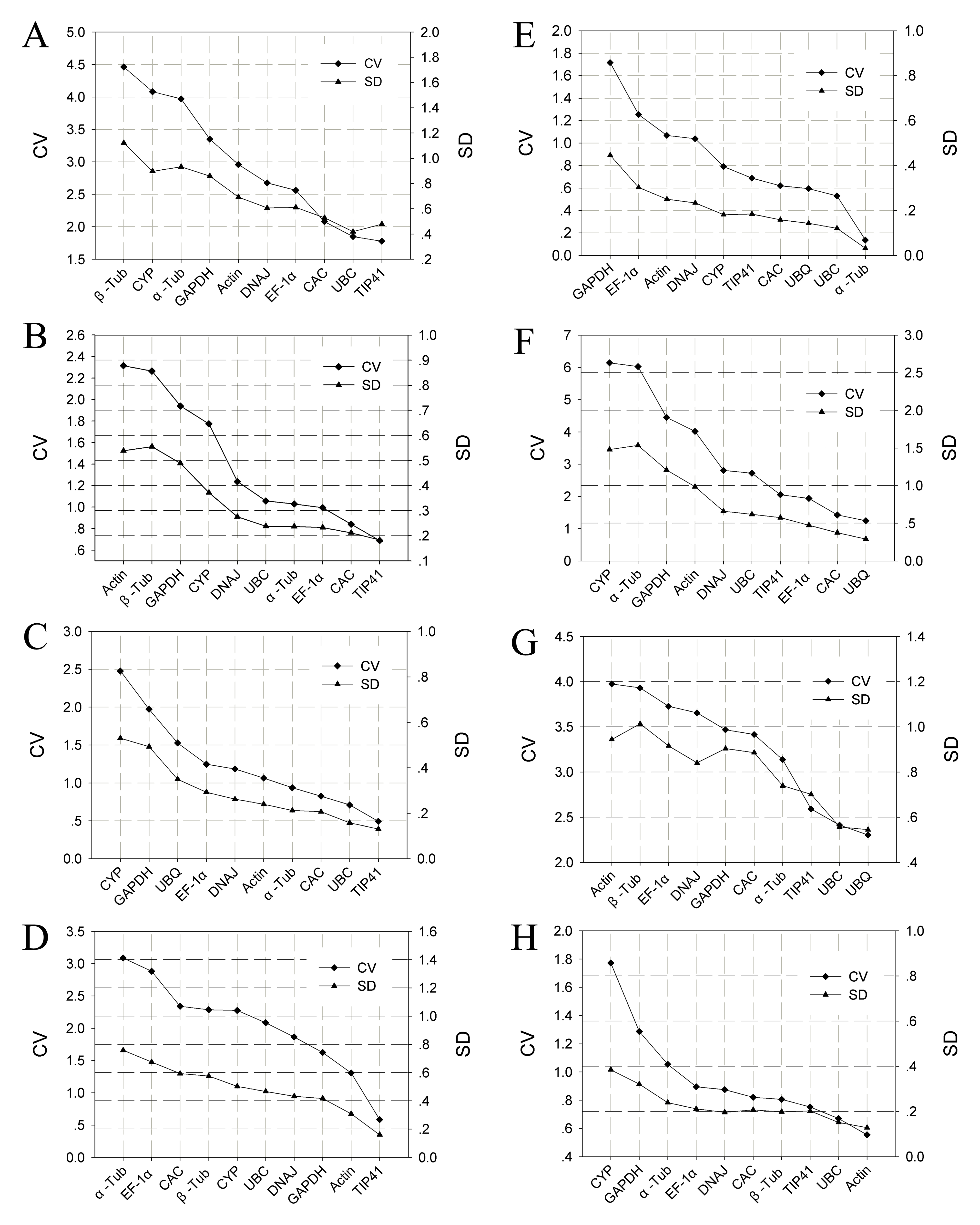

Supplement: Figure S5 — Stability ranking of candidate genes by BestKeeper. The gene with lower CV and SD shows more stable expression. CV: coefficient of variation; SD: standard deviation. (A) All Samples group; (B) NaCl group; (C) Nitrogen group; (D) Drought group; (E) Cold group; (F) Heat group; (G) Tissue group; (H) Age group. [file Image5.TIF]
